# LF‐EMF Compound Block Type Signal Activates Human Neutrophilic Granulocytes In Vivo

**DOI:** 10.1002/bem.22406

**Published:** 2022-04-28

**Authors:** Jan J. M. Cuppen, Cristian Gradinaru, Bregje E. Raap ‐ van Sleuwen, Anna C. E. de Wit, Ton A. A. J. van der Vegt, Huub F. J. Savelkoul

**Affiliations:** ^1^ Umani Medical BV Waalre The Netherlands; ^2^ Cell Biology and Immunology Group Wageningen University & Research Wageningen The Netherlands; ^3^ Bernhoven Hospital Uden The Netherlands

**Keywords:** neutrophils, infectious disease, innate immune system, LF‐EMF

## Abstract

This research aims to demonstrate in a randomized, placebo‐controlled crossover design study that a nominal 5 μT low‐frequency electromagnetic field (LF‐EMF) signal for 30 min activates neutrophils in vivo in humans. Granularity of neutrophils was measured in blood samples of healthy human volunteers (*n* = 32) taken before and after exposure for both the exposure and control sessions. A significant decrease in the granularity, indicative of neutrophil activation, was observed both in the exposure measurements and the exposure minus control measurements. Earlier EMF publications show immune function increase in isolated cells and more effective immune responses in animals with infections. This result, therefore, supports the thesis that the exposure can activate the innate immune system in humans, speed up the innate immune response, and may have potential beneficial effects in infectious disease. © 2022 Bioelectromagnetics Society.

## INTRODUCTION

Neutrophilic granulocytes are the most numerous white blood cells in humans (~2 billion in the bloodstream and 8–50 billion in tissue). They are first responders to a new infection. Neutrophils crucially provide early innate immune responses against a range of extracellular and intracellular bacteria but also control certain viral and fungal infections. Neutrophils display phenotypic and functional heterogeneity and regulate antimicrobial function and the innate immune response. An important recently discovered function is that neutrophils are able to activate and facilitate the response to an infection by other more specialized immune cells, and thereby connect the innate to the adaptive immune system [Breedveld et al., 2017]. As such, the activation state and speed of response of neutrophils have a big influence on reducing incubation time and the delay of the immune response to a new infection and thereby on the maximum size and extent (and damage caused) that the infection can attain.

Being highly motile, neutrophils quickly congregate at a focus of infection, attracted by cytokines and chemokines expressed by activated other cells (endothelium, mast cells, and macrophages). Neutrophils are recruited to the site of injury as early as within 15–30 min, and fully cover the affected area within approximately 4 h. Subsequently, neutrophils express and release cytokines, which in turn amplify inflammatory reactions by several other cell types and are therefore key in the front‐line defense against invading pathogens. Neutrophils have three methods for directly attacking micro‐organisms: phagocytosis (ingestion), degranulation (release of soluble anti‐microbials and cytokines), and generation of neutrophil extracellular traps (NETosis) [Segal, 2005; Ear and McDonald, 2008; Hickey and Kubes, 2009].

In addition to recruiting and activating other cells of the immune system, neutrophils are essential to the host defense against invading pathogens and fight constantly against different infections and toxic agents that enter via the skin, the respiratory, intestinal, urinary, and reproductive system, and the membranes of the eyes. These organs are normally lined by neutrophils, which help prevent the entry of organisms or foreign particles. A decreased availability and a reduced functional competence of neutrophils have been associated with a decreased immunocompetence and an increased susceptibility to infection [Moreira da Silva et al., 1994].

Degranulation, the secretion of neutrophil granules, is a critical effector function of neutrophils initiated early during neutrophil recruitment. The extent of neutrophil degranulation must be carefully balanced against the potential harm caused by the pathogen as the dysregulated release of proteolytic enzymes by neutrophils can degrade the extracellular matrix, contributing to immunopathology [Johansson and Kirsebom, 2021]. Balancing of antiviral responses in the lung is critical for managing the efficient clearance of the SARS‐CoV‐2 virus, while limiting tissue damage and avoiding compromising the lungs' ability to perform gas exchange. Moderate neutrophilia and controlled levels of NETosis are able to regulate the majority of pulmonary infections [Ackermann et al., 2021; Stegelmeier et al., 2021; Zuo et al., 2021].

Previous work has shown that a 30 min 5 µT exposure with a low‐frequency electromagnetic field (LF‐EMF) compound block type (CBT) signal can decrease mortality and tissue damage in animals with infections, and increase vitality [Cuppen et al., 2007]. It has also shown that the effect is uniformly present between 0.15 and 50 µT while reducing to zero below 0.01 µT [id.]. Reactive oxygen species (ROS) production was increased in freshly harvested monocytes [Kazemi et al., 2015], and NETosis was increased in neutrophilic granulocytes in human blood [Golbach et al., 2015]. The CBT signal is described in Bouwens et al. [2012] and below.

The goal of this research is to establish that neutrophils are activated in humans in vivo by the selected LF‐EMF exposure. To that end, we measure granularity of neutrophils in blood samples taken just before and immediately after the session. A decreased granularity thereby corresponds with a higher activation state of neutrophils [Bekkering and Torensma, 2013].

## MATERIALS AND METHODS

**Fig. 1:**
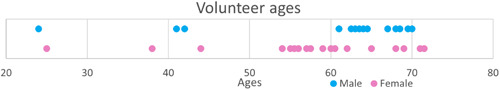
Age distribution of volunteers. The bias towards the 50–70 year age range is on purpose.

Healthy volunteers (*n* = 32) were recruited and invited to Bernhoven Hospital (Uden, The Netherlands) for two sessions at least 1 week apart. There were 18 female volunteers with mean age 57, SD 11, and 14 male volunteers with mean age 59, SD 13 (see Figure 1). This research was aimed to be a first step towards a treatment for preventing serious COVID‐19 after a SARS‐CoV‐2 infection. It is also a preparation for a trial with recurrent cystitis. Older people have a slower responding immune system and the majority of COVID‐19 patients are older men, while the majority of recurrent cystitis patients are older women. Therefore, we accepted volunteers with ages biased towards the older age brackets.

The sessions were identical except for the device choice, once exposure, and once control, in random order. Exclusion criteria that included COVID‐19, pregnancy, immune disease, or active implanted device were evaluated. Using telephone questionnaires after 1 day, 1 week, and 1 month after each session by a trained professional, no adverse events were reported by the participants.

The study protocol was approved by the Medical Ethical Committee of Radboud University Medical Center under number NL75559.091.20. Before starting treatment, 3 ml blood was taken and sent to the hospital lab, the 30 min treatment with the LF‐EMF device was performed, and afterwards another 3 ml blood was taken and also sent to the lab.

In the hospital lab, the samples were immediately analyzed in a normal hospital routine on an Abbott Cell‐Dyn Sapphire hematology analyzer (Abbott Diagnostics, Santa Clara, CA), which allows for whole‐blood analysis using spectrophotometry, electrical impedance, and laser light scattering (multi‐angle polarized scatter separation, MAPSS) to classify populations of blood cells. The device comprises detectors that are used for optical light scattering to measure different parameters that represent morphological characteristics (e.g. cellular volume, granularity, nuclear shape) of cells and based on combinations of these parameters, cells are automatically classified into different subpopulations, including neutrophils. This system stores blood count data (including neutrophil count NEU) and limited flow cytometric data (including the 7‐degree forward scatter measurement Nimn (technical code) indicating granularity) which were used in the analysis.

**Fig. 2:**
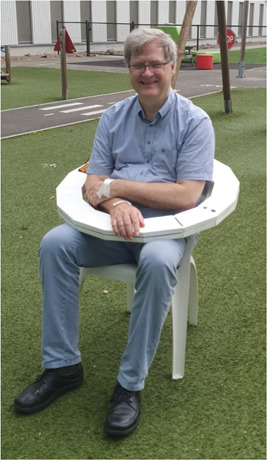
Photo of the exposure setup. The device is ring‐shaped, lightweight, and runs on a battery pack of 10 AA NiMH batteries.

Two treatment systems were provided, one exposure, one control, marked A and B on the bottom. The coding (A and B being exposure and control or vice versa) was chosen by coin flip and not revealed to operators. Volunteers did not even see the markings. The statistician that performed the analysis received the coding resolution only after the analysis. The devices contained custom electronics that operated on a removable battery pack with 10 standard AA 1.2 V NiMH batteries which were charged to full capacity before each session. A button started the treatment which stopped after 30 min as indicated by a light‐emitting diode in the button. A ring‐shaped coil system was provided that was positioned exactly around the volunteer as shown in Figure 2. All volunteers were positioned the same: facing the earth magnetic South. This caused the plane of the ring to be substantially perpendicular to the earth magnetic field direction, which is 60° downwards in The Netherlands, and the direction of the exposure field to be substantially parallel to it. The location was always the same spot in the hospital children's garden, outside, and the earth magnetic field strength was 47 µT, measured with an FW Bell 5170 magnetometer (OECO, Milwaukie, OR).

**Fig. 3:**
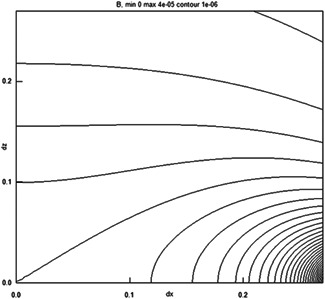
27 cm × 27 cm Homogeneity plot of the exposure field. 5 µT contour runs through origin, contour spacing is 1µT. The plot shows the *xz* rectangle; there is circular symmetry around the *z*‐axis and symmetry around the *z* = 0 plane.

The field strength implemented is 5 µT rms in the center of the coil for a coil current of 0.88 A in the coil with 2 0.75 mm^2^ copper windings. Off center in the coil plane, the field strength increases to about 50 µT close to the coil cover at 28 cm radius. Away from the coil plane, the field strength decreases approximately linearly along the *z*‐axis by 1 µT per 8 cm for the first 30 cm. A homogeneity plot 27 × 27 cm is given in Figure 3. Please note that the homogeneity is circularly symmetric around the *z*‐axis. The 5 µT contour is the one through the origin; contour spacing is 1 µT. Forces on the windings, predominantly radial, are less than 42 µN, i.e. 4 mg, and heat generated is less than 0.5 W, both integrated around the complete circumference. No detectable sound, vibration, or heating is generated at this exposure field strength.

The device and the exposure can be considered safe as the exposure remains within all safety guidelines set by the International Commission on Non‐Ionizing Radiation Protection [ICNIRP, 2003] that are adopted by the WHO and EU. The CBT signal is generated from a summation of block waves, centered around 0, with frequencies 320, 730, 880, and 2600 Hz. The ramps were slanted so that the blocks became trapezoids with 10 µs transition time. We have optimized our electronics to faithfully create the ramps in the CBT signal while allowing low frequency (i.e. low *dB*/*dt*) deviations. Because block transitions were separated in software, the maximum *dB*/*dt* was 2 T/s at the periphery and 0.2 T/s in the center of the coil. Models developed by Pilla [2003] imply that the bioactive parts of the signal are the high *dB*/*dt* parts.

The reasons for the choice of design parameters for exposure ‐field, ‐procedure, and ‐coil are that (i) previous research showed significant and useful effects in animals and on cell level between 0.15 and 50 µT [Cuppen et al., 2007] and (ii) these parameters allow for design of a simple, portable device that could be used in clinical practice including home use, if in the future the method can also be proven for disease mitigation.

## STATISTICS

All statistical analyses were performed using Stata/IC v15.1 (StataCorp, College Station, TX). The power studies were performed using G*Power 3.1.9.4 (HHU, Düsseldorf, Germany). Data normality tests were performed visually using QQ plots and then confirmed quantitatively by means of the Shapiro–Wilk test. The Shapiro–Wilk test returns a nonsignificant result for both groups (*P* = 0.137, Group A; *P* = 0.191, Group B), as well as for A–B (*P* = 0.285), which indicates that the null hypothesis of normally distributed data cannot be rejected for Group A, Group B, nor for the difference data in Group A–B (the ΔΔNimn variable defined below).

Hence, a one‐sided paired *t* test was used to test the null‐hypothesis that the population mean for ΔNimn for Groups A and B is zero (i.e. no difference between before and after treatment) against the alternative that a decrease in Nimn occurs after treatment (ΔNimn < 0). This was done for the 23 volunteers in Group A and independently for the 28 volunteers in Group B (total number of volunteers is 23 + 28 − 19 [counted double] = 32).

Additionally, since the main aim of the study is to test the difference between control and exposure, a paired analysis was also performed using the 19 volunteers which appear in both Groups A and B. In this case, the primary analysis null‐hypothesis will be that ΔNimn is equal between Groups A and B, or equivalently the ΔΔNimn variable (defined as the difference between ΔNimn in Group B and ΔNimn in Group A for paired subjects) is equal to zero. The alternative hypothesis is that the exposure results in a more significant decrease of ΔNimn than control (ΔΔNimn > 0).

Using a linear model, it was established that the order of treatment (exposure first or control first with at least 1 week in between) does not affect the outcome, i.e. the coefficient of the order of treatment in the model is not statistically significantly different from zero (*P* = 0.523). Hence, a one‐sided paired *t* test can be used to compare ΔNimn between Groups A and B. The null hypothesis is that ΔNimn(A) = ΔNimn(B), and the alternative is that ΔNimn(A) > ΔNimn(B).

## RESULTS

**Fig. 4:**
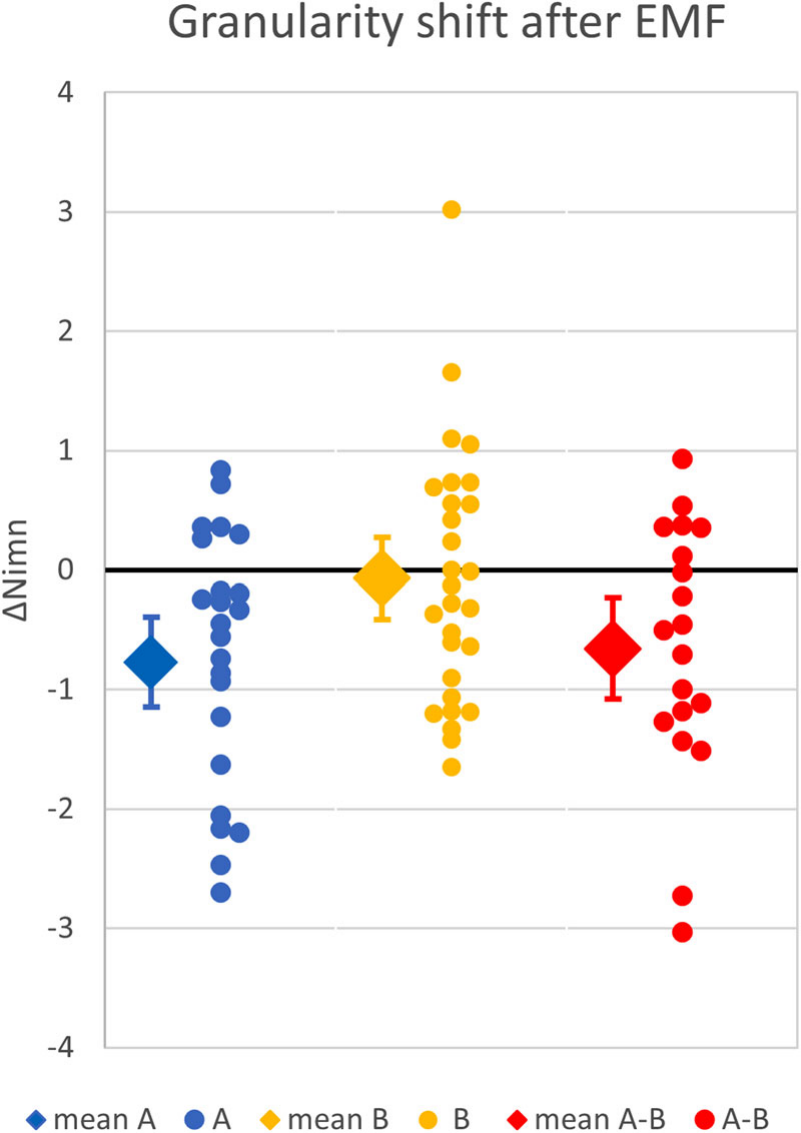
Results. Granularity measured in peripheral blood after exposure minus before exposure. In each group to the left mean and *P* = 0.05 confidence interval, next data points of individual results. Left group A exposure, middle B control, right Groups A–B for the same volunteer at least one week between exposures. Decreasing granularity indicates neutrophils excrete antimicrobial proteins and mediators for further immune response [Bekkering and Torensma, 2013].

After statistical analysis, it was revealed that Group A was exposure and Group B was control exposed. The baseline value of Nimn (arbitrary units) in this experimental setup was around 140. The data of the calculated granularity shift after EMF exposure are presented in Figure 4. On the left (in blue) the exposure results are shown that display a significant (*P* = 0.0019) decrease in granularity with a mean = − 0.71 and SD = 1.05 after EMF exposure. The applicable mean and confidence interval (0.38 for *P* = 0.05) are shown to the left of the datapoints. The achieved power is 93%.

The control set of granularity datapoints (middle, in yellow) is not significantly shifted after EMF exposure. Mean = − 0.07, *P* = 0.37, confidence interval (*P* = 0.05) = 0.34.

The analysis for A–B or $∆{∆\mathrm{Nimn}}_{i}={∆\mathrm{Nimn}}_{i}\left(B\right)-{∆\mathrm{Nimn}}_{i}(A)(\text{in red})$ yields that the observed effect on granularity shift after EMF exposure is significant with *P* = 0.008 and achieved power 82%. The confidence interval is 0.42 (*P* = 0.05).

**Fig. 5:**
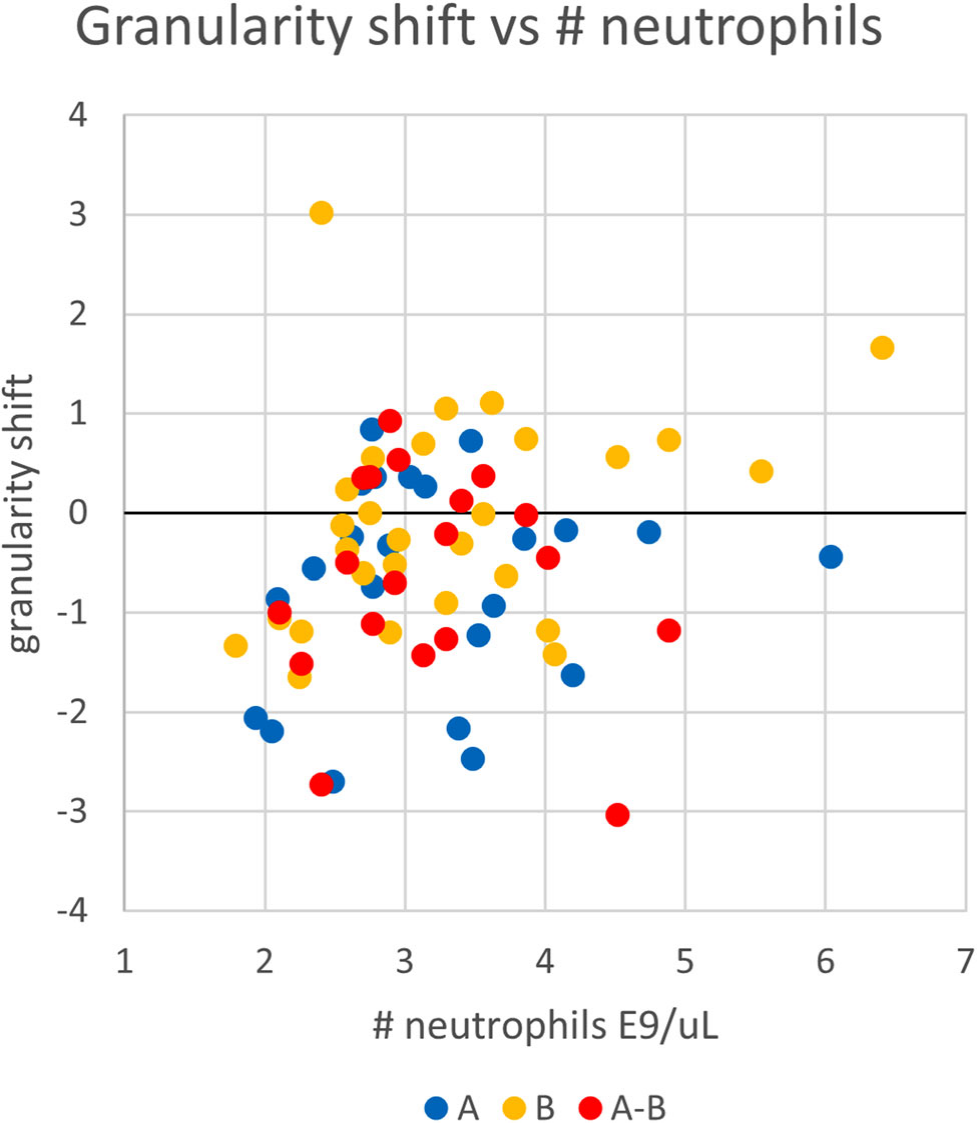
Granularity shift versus neutrophil count; with a correlation coefficient *R* = 0.165 in the A group and *R* = 0.365 in the B group, there is no apparent correlation between the granularity shift and the relative number of neutrophils per unit volume in the sample. This suggests that there is no correlation between the immune competence of the volunteer and the granularity shift.

With a correlation coefficient *R* = 0.165 in the A group and *R* = 0.365 in the B group, there is no apparent correlation between the granularity shift and the relative number of neutrophils per unit volume in the sample as is visible in Figure 5. This implies that there is no apparent correlation between the granularity shift (measured in the Nimn parameter) and the immune competence (measured as neutrophils per unit volume) of the volunteer.

## DISCUSSION

Our results indicate that a short‐term low dose LF‐EMF exposure in vivo indeed has a significant activating effect on neutrophils in the peripheral blood of human volunteers. Consequently, the animal and in vitro human cell level effects that were found for the CBT signal in our previous studies [Cuppen et al., 2007; Golbach et al., 2015] are indicative that similar results could be achieved in vivo. These earlier studies showed a strongly decreased mortality of infected and/or diseased animals, significantly reduced tissue damage, increased vitality, and increased functional response of immune cells after infection or a simulated infection (with LPS in in vitro cell culture experiments). These results now set the stage for further investigation of immune activation by LF‐EMF in humans in actual disease situations in vivo.

Our study showed that LF‐EMF exposure in vivo in clinically healthy individuals results in rapid and detectable activation of neutrophils in peripheral blood based on established activation markers like decreased granularity [Bekkering and Torensma, 2013]. Initial activation of neutrophils results in the induction of a priming by exposure to early phase mediators and leaving the neutrophils endowed with a more rapid functional response while staying in a resting condition. This response will be more rapid and amplified upon another stimulus, including exposure to an infection. Since neutrophils contribute to the production of type I IFN [Shirafuji et al., 1990], they prime the innate immune system to persist in a preactivated antiviral and antibacterial state, associated with further large secretion of type I IFN from plasmacytoid dendritic cells [Pierce et al., 2021]. Increased expression of genes downstream of type I and II IFNs and the NLR family pyrin domain‐containing 3 (NLRP3) inflammasome results in a more vigorous and faster early immune response against infections, including SARS‐CoV‐2, and thereby mitigates the development of severe Covid‐19 disease [Pylaeva et al., 2016; Loske et al., 2022; Chou et al., 2022]. Similarly, a preactivated bacterial state can be expected to mitigate diseases like cystitis.

As discussed above, activated neutrophils can also cause tissue damage. It is our expectation that future research will show that early in a serious infection, the advantages of a faster and stronger immune response far outweigh the disadvantages because both the maximum infection and the maximum immune response can remain within boundaries of regulation and will therefore be more optimally controlled.

The field strength used in the exposure is well below all safety guidelines adopted by the WHO and EU [International Commission on Non‐Ionizing Radiation Protection [ICNIRP], 2003]. In fact, physicists have long maintained that such a low field strength EMF cannot achieve biological effects because the energy deposited is negligible. From in vitro studies, it is known that even minor exposure to LF‐EMF can have effects on cellular mechanisms [Mahaki et al., 2019]. Indeed, it was found that cells are responsive to field strengths as low as ~0.15 µT [Kapri‐Pardes et al., 2017]. It was proposed that NADPH oxidase (NOX) acts as a receptor for LF‐EMF [Golbach et al., 2015; Kapri‐Pardes et al., 2017] and subsequent activation of NOX generates ROS. In vitro, neutrophils display increased ROS production already after 15 min of exposure to EMF [Poniedzialek et al., 2013].

The question ‘how and why cells detect low strength LF‐EMF signals and respond to them’ is beyond the scope of this article, although there is abundant evidence that they do [Rosado et al., 2018]. Resonance effects probably play a role, for which a 30 min continuous exposure apparently is enough. Previously, we analyzed the effect of LF‐EMF exposure on the expression of inflammatory genes and proteins in monocytes but did not find consistent effects, and we, therefore, focused on neutrophilic granulocytes as they are more abundant and more agile leukocytes that do contain granules, thereby enabling the measurement of the density shift reported here [Bouwens et al., 2012; Loske et al., 2022]. Although the effect we measured is subtle (a shift of 0.7 on a baseline of 140), a downstream effect on immune response as in our previous animal experiments [Cuppen et al., 2007] can be explained by the fact that the body contains billions of neutrophils, of which only a few need to be sufficiently activated. Neutrophils display various robust positive feedback amplification processes that steer effective migration towards chemotactic gradients, induce multiple waves of neutrophil recruitment to the site of inflammation and infection, and promote various effector functions of other cells to provide antimicrobial immunity [Németh and Mócsai, 2016]. When only a few neutrophils engage and recognize such a pathogen, they will start a cascade of activation and amplification of neutrophils (as they are so abundant with about 60% of peripheral blood leukocytes) and other cells such as monocytes, that provide an efficient innate immune response [Francis et al., 2019].

The short‐term exposure, of which possible effects can be rapidly detected, allowed for a randomized, placebo‐controlled crossover design which could be completed in a short time. The primary outcome variable was the activation state of the neutrophils that could be determined in a routine setting in the clinical chemistry laboratory of a hospital. Several confounding factors, however, have to be considered, as this could help avoid loss of samples as happened in our study due to these unforeseen issues. First, different Sapphire analyzers, even when duly calibrated to give precise blood cell counts, have a different baseline for Nimn. Second, due to the high sensitivity of neutrophils to environmental conditions, a delay in analyzing after blood sampling induced substantial shifts in Nimn. Therefore, all samples must be analyzed as soon as possible, and delays should be avoided. Third, using tubes, there might be a risk on blood sample coagulation which requires drawing an extra blood sample. Also, inside (hospital) buildings the ambient static field can be significantly changed in direction and strength due to construction iron. Moreover, when working in an area (or building) with an earth (or ambient) magnetic field strength different from The Netherlands (47 µT), the frequencies may have to be changed (linearly) to achieve a similar resonance potential. Lastly, it is crucial to maintain sufficient distance between individuals receiving the exposure and the control exposures, as even very small signals (down to 3 nT) can still have an effect on neutrophils [Cuppen et al., 2007].

## CONCLUSION

The results of this study indicate that the LF‐EMF treatment activates neutrophils in vivo in humans, aged between 50 and 70. Therefore, LF‐EMF exposure with the CBT signal may have the potential to be applied in providing a treatment modality in infectious diseases, especially when applied early in the infection by speeding up the immune response that could keep the infection smaller and reduce its clinical effects on the patient.

## AUTHOR CONTRIBUTIONS

JC originally identified immune stimulation with EMF as a research subject inspired by work by Dr. Georges Vanroy (†2003) in chronic infectious disease using combined electric and electromagnetic stimuli. JC designed the signal, the device hardware and software, and designed and supervised the study. HS originally identified neutrophils as a target for research in EMF immune stimulation and the granularity measurement as a method of interest. CG did the statistics, and BR coordinated and managed the data collection with support from AW. JC and HS wrote the article. TV and JC declare to own stock in Umani Medical BV, which owns the IP concerning the method and devices used. CG and AW are coworkers of Umani Medical BV.
